# Contributing and limiting factors to guideline-adherent therapy in senior and elderly breast cancer patients: a questionnaire-based cross-sectional study using clinical and cancer registry data in Germany

**DOI:** 10.1007/s00432-023-05446-y

**Published:** 2023-10-10

**Authors:** Andreas Heidenreich, Rabea Fuchshofen, Susanne Elsner, Frank Gieseler, Alexander Katalinic, Joachim Hübner

**Affiliations:** 1 Institute of Social Medicine and Epidemiology, University of Luebeck, Ratzeburger Allee 160, 23560 Luebeck, Germany; 2 Clinic for Hematology and Oncology, University Hospital Schleswig‐Holstein (UKSH), Luebeck, Germany; 3 Agency for Clinical Cancer Data of Lower Saxony, Oldenburg, Germany

**Keywords:** Breast cancer, Guidelines, Patient needs, Patient preferences

## Abstract

**Purpose:**

Elderly cancer patients are less likely to be treated in accordance with evidence-based guideline recommendations. This study examines patient-related factors associated with deviations from guideline recommendations.

**Methods:**

Using medical documentation and cancer registry data, we investigated the treatment courses of female breast cancer patients aged 50 and older in Germany regarding compliance with German guidelines. Participants completed a questionnaire querying factors hypothesized to be associated with guideline adherence. We conducted univariate analyses to explore the data and select variables for multivariate logistic regression to estimate adjusted odds ratios.

**Results:**

Of 1150 participants, 206 (17.9%) were treated in deviation from guideline recommendations. Patients 70 years and older were more likely to be treated deviating from guideline recommendations than patients 50–69 years old (OR: 2.07; 95% CI: 1.52–2.80). Patients aged 50–69 years who reported that quality of life guided their treatment decision were more likely to be treated in deviation from guideline recommendations (AOR: 2.08; 95% CI: 1.11–3.92) than the elderly. In older patients, higher age was associated with an increased chance of receiving guideline-discordant care (AOR: 1.06; 95% CI: 1.01–1.11), as was depression diagnosed prior to cancer (AOR: 1.84; 95% CI: 1.00–3.40).

**Conclusion:**

Reasons for deviations from guideline recommendations in breast cancer patients differ by age. In decision-making concerning elderly patients, particular attention should be paid to those with pre-existing depressive disorders. Adequately addressing their needs and concerns could prevent inappropriate deviations from guideline recommendations.

## Introduction

Breast cancer remains the most common cancer in Germany (Robert Koch Institute 2022). As age demographics shift and patients live longer, increased attention must be paid to their unique characteristics and needs (Gomes et al. 2020). Guidelines have partly taken up the topic of ageing, as in the case of Germany’s “S3” guideline (“step 3” indicating the highest level of empirical evidence and expert consensus) for breast cancer (Leitlinienprogramm Onkologie 2017). Special needs of elderly patients are touched upon therein but rarely are age-specific treatment recommendations given.

Preliminary work has shown that older patients with cancer (70 years and older) are more often treated with deviation from guideline recommendations than younger patients. Older breast cancer patients are less likely to receive breast conservation therapy, adjuvant therapy, or radiation (Waldmann et al. 2008; Hancke et al. 2010; Peters et al. 2015; Wallwiener et al. 2016). It has also been shown that deviations from specific guideline recommendations are associated with higher mortality or lower recurrence-free survival in older patients (Wöckel et al. 2010). The interpretation of this finding is not straightforward, as various factors influence the treatment decision and its success. For example, medical factors, such as diminished organ functions, may stand in the way of guideline-congruent therapy. In addition to medical factors, many other conceivable aspects can cause therapy to deviate from the guideline recommendations. If, for example, a guideline-congruent treatment option is not pursued due to the patient’s values or individual preferences, then a deviation from the corresponding recommendation is not deficient but desirable in the sense of patient-oriented care, provided that this decision was made in an informed manner and with consideration of the evidence. Deviation from guideline recommendations is particularly problematic when due to poor communication about personal opinions and preferences. These considerations touch on fundamental questions about the “sense” or “meaningfulness” of specific therapy options concerning the patient’s life situation. Doctors, patients, or relatives can influence the therapy decision with such assessments (Gieseler et al. 2021). What is considered “still reasonable” for the older patient may deviate from the guideline recommendations. There is insufficient knowledge about the underlying factors contributing to this discrepancy and the general reasons for non-guideline compliant treatment. A recent study examined facilitators of and barriers to guideline-concordant care of breast cancer patients in the United States (McDougall et al. 2021). The authors identified social support, patient support services, and communication issues as intervention targets for improving breast cancer care. However, the authors enrolled patients aged 26 to 69, and transferring the findings to older populations is not readily admissible. Furthermore, their study did not address patients’ attitudes and preferences, and aspects of medical communication and cooperation were not dealt with in depth, although those were repeatedly identified as issues that complicate the treatment process and cause a lack of comprehension of medical knowledge (Kruse 2013; Füeßl 2015; van Vliet et al. 2015; Hoffmann et al. 2018; Groen-van de Ven et al. 2018; Jack et al. 2019). Therefore, we conducted a questionnaire-based cross-sectional study using clinical and cancer registry data in Northern Germany, analyzing clinical data and querying seniors (50–69 years at diagnosis) and elderly (70 years at diagnosis) breast cancer survivors, covering attitudes and preferences in addition to physical and psychosocial factors.

## Methods

### Study population and recruitment

We recruited female breast cancer patients (ICD-10 C50) at least 50 years old at the time of initial diagnosis via six cooperating hospitals in Germany and the cancer registries of the federal states of Hamburg and Schleswig–Holstein. Patients in hospitals were enrolled between December 2018 and July 2019 during their inpatient stay. They were handed an initial questionnaire (not part of this analysis) and received a follow-up questionnaire after 6 months when primary therapy was presumed to be completed. Additionally, eligible patients were identified in the cancer registries and invited by postal mail to participate in the study and were sent the questionnaire. Patients contacted through the cancer registries received their initial breast cancer diagnosis between January 2018 and December 2019.

Written, informed consent for the usage of questionnaire data and insight into relevant medical documentation or data from cancer registries, respectively, was sought from all participants. The University of Luebeck’s ethics committee has approved the study’s conduct (decision: October 5, 2017; file: 17–288).

### Operationalization of guideline adherence

We defined guideline adherence based on the German evidence- and consensus-based (“S3”-level) breast cancer guidelines, version 4.0 (Leitlinienprogramm Onkologie 2017; Wöckel et al. 2018a, b). The recommendations set out therein were applicable at the time of the therapies of all participants. Of the numerous recommendations made therein, we chose four essential recommendations that are highly clinically relevant and could be reliably operationalized with the available data. We considered recommendations on breast-conserving surgical treatment (guideline reference number: 4.20), adjuvant radiotherapy after breast-conserving surgical therapy (4.36), endocrine therapy for estrogen and/or progesterone receptor-positive tumors (4.50), and adjuvant systemic therapy for HER2-positive and triple-negative tumors (4.55).

Data sought included information on age, sex, date of diagnosis, TNM, tumor localization, therapies conducted (form, beginning and end), and administered therapeutics. We obtained excerpts from the clinical information systems of the participating hospitals electronically or in paper form and received electronic data sets from the cancer registries upon application to the competent authority. We used these data to determine whether the therapy documented therein aligned with relevant guideline recommendations. The patient’s therapy was classified as “non-adherent” for the primary outcome measure if the treatment deviated from at least one relevant recommendation. When the medical data lacked crucial details for determining guideline adherence or was inconclusive, we queried patients’ survey responses for information on therapies considered, carried out, or discontinued to fill in gaps, where applicable.

### Survey data

We developed a questionnaire to collect responses on multiple dimensions potentially relevant to the therapy decision and its concordance with guideline recommendations, as well as the patient’s subjective judgment of whether the therapy carried out was appropriate for their situation. Dimensions encompassed physical factors and comorbidities, social participation and support, and subjective views, judgments, and knowledge. We used established instruments to assess frailty [Tilburg Frailty Indicator, TFI (Freitag et al. 2016)], self-efficacy beliefs [Short Scale for Measuring General Self-efficacy Beliefs, ASKU (Beierlein et al. 2014, 2017)], and social support [Questionnaire on Social Support, FSozU-14 (Fydrich et al. 2009)]. Questionnaire items on Likert scales with five response options (“strongly disagree” to “strongly agree”) were dichotomized between the neutral response option and “agree” to establish whether “agreement” or “no agreement or disagreement” to a statement influences the target outcome. Satisfaction with the therapy decision-making process was assessed using a seven-point Likert scale. Dichotomization was done using a median split. Information on survey items used is displayed in Table 1.

**Table 1:** Overview of survey items

Dimension	Item	Operationalization
Patient-related factors	Frailty	Tilburg Frailty Indicator, dichotomized per instrument instructions (≥ 5)
	Need of care	“Pflegegrad” 1–5 according to German social code § 15 SGB XI and “No”, dichotomized to reflect existence of “Pflegegrad”
	Depression diagnosed before cancer diagnosis	Dichotomous
	Regular intake of four or more medications	Dichotomous
	Independently mobile by access and ability to use car or public transport	Dichotomous
	Social support	FSoZU social-support scale, continuous
	Confidant available for advice on therapy decision	Dichotomous
	Self-efficacy expectations	ASKU self-efficacy scale, continuous
	Precedence of quality of life or prolongation of life in therapy decision	Answer options “both equally important”, “prolongation of life”, “quality of life”
	“I have many expectations from life and wish that I can experience many more years”	5-point Likert scale, “strongly disagree” to “strongly agree”; dichotomization reflects agreement to statement
	“I have tasks in life and wish that I can fulfil them for a long time”	5-point Likert scale, “strongly disagree” to “strongly agree”; dichotomization reflects agreement to statement
Decision-related factors	“All in all, how satisfied are you with how treatment decisions were made?”	7-point Likert scale, “very dissatisfied” to “very satisfied”; dichotomized along 6 points
	“I was well-informed about my illness and treatment options.”	5-point Likert scale, “strongly disagree” to “strongly agree”; dichotomization reflects agreement to statement
	“I was well-included in treatment decisions.”	5-point Likert scale, “strongly disagree” to “strongly agree”; dichotomization reflects agreement to statement
	“Do you know medical guidelines or patient guidelines for treating breast cancer?”	Answer options “don’t know”, “know but didn’t read”, “read it”; dichotomization reflects reading
	Psychological consultation offered	Dichotomous
	Empathy	Dichotomous
Retrospective judgment of treatment decision	“I am sure that the therapies carried out were appropriate for my situation”	Dichotomous

### Statistical analysis

We characterized the study sample with descriptive statistics by cancer staging and medical factors separately by age group: 50–69 years (senior patients) and 70 years and older (elderly patients). We used Fisher’s exact tests to analyze expected and observed frequencies. We examined the proportions of participants treated deviating from each guideline recommendation by age group. We used univariate logistic regression to estimate odds ratios (OR) for covariates concerning the outcome measure and to identify variables of interest associated with deviations from guideline recommendations. Covariates with *P* values of < 0.1 were entered in separate multivariate logistic regression models for each age group to estimate adjusted odds ratios (AOR) and 95% confidence intervals (CI). Within these models, associations were accepted based on a significance level of 0.05 (5%). We tested the models for multicollinearity using generalized variance inflation factors [GVIF^(1/(2df))^ (Fox and Monette 1992)]. All statistical analysis was performed with the *R* statistical software, version 4.2.2 (R Core Team 2022).

## Results

Out of 3075 patients contacted via the cancer registries of Hamburg and Schleswig–Holstein, 1315 (42.8%) returned a questionnaire—590 (32.7%) and 725 (57.1%), respectively. Responders were slightly younger than non-responders (66.1 years vs 68.6 years; *P* < 0.001*)*. On average, questionnaires from patients recruited via cancer registries were answered 79 weeks after diagnosis; this was 34 weeks for patients recruited via cooperating clinics. Overall, 1,112 responders from cancer registries and 38 patients recruited in cooperating clinics submitted a usable questionnaire, and sufficient clinical data were available to assess at least one relevant guideline recommendation. Participants were predominantly diagnosed in earlier stages of cancer, with UICC stage I being most commonly observed in both senior and elderly patients (66% and 51%, respectively). Elderly cancer patients were more frequently in need of care (OR: 4.56; 95% CI: 2.56–8.44) and more likely to take more than four medications (OR: 1.81; 95% CI: 1.39–2.35) but were less frequently diagnosed with depression compared to younger patients (OR: 0.46; 95% CI: 0.33–0.64). The proportion of frail patients does not differ significantly by age group (OR: 1.68; 95% CI: 0.91–1.50). Patient characteristics are displayed in Table 2.

**Table 2:** Overview of patient characteristics

	50–69 years, *N* = 698	70 years and older, *N* = 452
Age at breast cancer diagnosis		
Median (IQR)	60 (55, 65)	77 (73, 80)
Period between diagnosis and survey response (weeks)		
Median (IQR)	78 (58, 101)	78 (54, 103)
UICC		
I	408 (66%)	208 (51%)
II	176 (28%)	159 (39%)
III	23 (3.7%)	32 (7.8%)
IV	15 (2.4%)	12 (2.9%)
Physical aspects and comorbidities		
Depression diagnosed prior to cancer diagnosis		
No	449 (73%)	354 (85%)
Yes	168 (27%)	61 (15%)
Regular intake of four or more medications		
No	522 (75%)	279 (62%)
Yes	175 (25%)	169 (38%)
Frailty (Tilburg Frailty Indicator)		
No	390 (58%)	225 (54%)
Yes	285 (42%)	192 (46%)
Need of care		
No	672 (97%)	393 (89%)
Yes	18 (3%)	48 (11%)

**Fig. 1:**
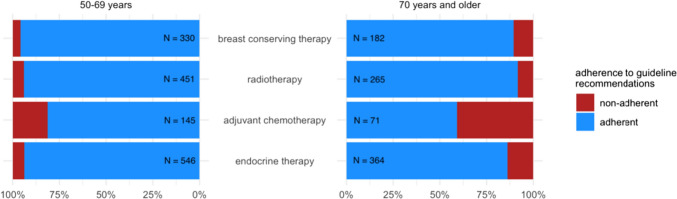
Proportion of patients treated in accordance with guideline recommendations per age group

Deviations from guideline recommendations were observed in 206 (17.9%) patients, with 95 (13.6%) in the younger age group vs 111 (24.6%) in the elderly (OR: 2.07; 95% CI: 1.52–2.80). In both younger and elderly patients, deviation from guideline recommendations was associated with the subjective notion of not having received treatment suitable to their individual situation (OR: 0.48; 95% CI: 0.27–0.87 and OR: 0.52; 95% CI: 0.29–0.95, respectively). Of patients for whom more than one guideline recommendation was relevant, 3.2% in the age group 70 years and older were treated in deviation from the guideline recommendations in more than one manner. In the age group of 50–69 years, this was 1.5% (OR: 2.24; 95% CI: 0.82–6.08) (see Table 3). The guideline recommendation for adjuvant chemotherapy for HER2-positive or triple-negative tumors was the most frequently not followed in both age groups, with a non-adherence rate of 18.6% in younger patients and 40.8% in older patients (OR: 3.02; 95% CI: 1.60–5.67). Proportions of guideline adherence per recommendation differentiated by age groups are displayed in Fig. 1.

**Table 3:** Number of deviations from guidelines in patients eligible for treatment respective to more than one guideline recommendation

	50–69 years (*N* = 477)	70 years and older (*N* = 283)
No deviation	402 (84.3%)	208 (73.5%)
One deviation	68 (14.3%)	66 (23.3%)
Two and more deviations	7 (1.5%)	9 (3.2%)

The univariate analysis of potential explanatory variables showed that different factors were associated with adherence to guideline recommendations depending on the age group. Depression before cancer diagnosis was linked with deviations from guideline recommendations in older patients (OR: 1.80; 95% CI: 1.00–3.22). The other variables assessing comorbidities (frailty, intake of medications, and need for care) did not significantly affect adherence to guideline recommendations in any age group. Conversely, for the younger age group, independent mobility was associated with adherence to guideline recommendations (OR: 2.98; 95% CI: 1.01–8.78). While the patient’s age at diagnosis did not matter in the age group 50 to 69 years, it was a significant factor in the group of elderly patients. Therein, older age reduces the chance of treatment according to the guidelines (OR: 0.94; 95% CI: 0.90–0.99). The satisfaction with the therapy decision-making process was significantly associated with guideline-adherent therapy in the elderly (OR: 0.52; 95% CI: 0.33–0.83). The associations between adherence to guideline recommendations and potential explanatory variables, differentiated by age groups, are shown in Table 4.

**Table 4:** Univariate regression analysis of explanatory variables by adherence to guideline recommendations

	50–69 years					70 years and older				
Characteristic	*N*	Non-adherent	OR^1^	95% CI^1^	*P*	*N*	Non-adherent	OR^1^	95% CI^1^	*P*
Age at diagnosis in years	698	95	0.99	0.95, 1.03	0.620	452	111	1.06	1.01, 1.11	**0.010**
UICC					0.275					0.396
I	408	67	–	–		208	55	–	–	
II	176	20	0.65	0.38, 1.11		159	45	1.10	0.69, 1.74	
III	23	2	0.48	0.11, 2.12		32	6	0.64	0.25, 1.64	
IV	15	1	0.36	0.05, 2.81		12	1	0.25	0.03, 2.00	
Frailty					0.383					0.800
No	390	57	–	–		225	55	–	–	
Yes	285	35	0.82	0.52, 1.28		192	49	1.06	0.68, 1.65	
Depression					0.930					**0.049**
No	449	60	–	–		354	80	–	–	
Yes	168	22	0.98	0.58, 1.65		61	21	1.80	1.00, 3.22	
Medication					0.637					0.911
No	522	73	–	–		279	69	–	–	
Yes	175	22	0.88	0.53, 1.47		169	41	0.97	0.62, 1.52	
Need of care					0.766					0.380
No	672	91	–	–		393	92	–	–	
Yes	18	2	0.80	0.18, 3.53		48	14	1.35	0.69, 2.62	
Mobility					**0.048**					0.525
No	16	5	–	–		35	7	–	–	
Yes	680	90	0.34	0.11, 0.99		411	102	1.32	0.56, 3.11	
Social support	692	91	0.97	0.71, 1.34	0.872	437	103	1.00	0.72, 1.37	0.982
Confidant available					**0.056**					0.127
No	72	15	–	–		51	17	–	–	
Yes	619	78	0.55	0.30, 1.01		392	92	0.61	0.33, 1.15	
Self-efficacy expectation	698	95	1.02	0.82, 1.27	0.836	452	111	0.91	0.72, 1.14	0.399
Prolongation vs quality of life?					**0.093**					0.939
Both equally important	557	70	–	–		335	76	–	–	
Prolongation of life	57	7	0.97	0.42, 2.23		12	3	1.14	0.30, 4.30	
Quality of life	73	16	1.95	1.06, 3.59		71	15	0.91	0.49, 1.70	
Expectations from life					0.313					0.512
No	55	5	–	–		92	25	–	–	
Yes	636	89	1.63	0.63, 4.19		352	84	0.84	0.50, 1.41	
Tasks in life					0.934					0.246
No	94	13	–	–		98	28	–	–	
Yes	592	80	0.97	0.52, 1.83		341	78	0.74	0.45, 1.23	
Satisfied with decision-making					0.127					**0.006**
No	197	33	–	–		120	40	–	–	
Yes	487	60	0.70	0.44, 1.11		320	66	0.52	0.33, 0.83	
Knowledge of guidelines					**0.068**					0.174
Did not read	351	56	–	–		253	67	–	–	
Read it	324	36	0.66	0.42, 1.03		178	37	0.73	0.46, 1.15	
Psychological consultation offered					**0.080**					0.162
No	220	37	–	–		239	65	–	–	
Yes	470	56	0.67	0.43, 1.05		205	44	0.73	0.47, 1.13	
Informed about illness and therapy					**0.064**					0.944
No	108	21	–	–		46	11	–	–	
Yes	582	74	0.60	0.35, 1.03		402	98	1.03	0.50, 2.10	
Physicians were understanding					0.642					0.307
No	364	52	–	–		237	63	–	–	
Yes	329	43	0.90	0.58, 1.39		214	48	0.80	0.52, 1.23	
Included in decision processes					0.844					0.240
No	206	29	–	–		105	30	–	–	
Yes	481	65	0.95	0.60, 1.53		340	78	0.74	0.45, 1.22	

^1^OR = Odds Ratio, CI = Confidence Interval, and bold *P*values (< .1) indicate subsequent inclusion in multivariate regression

In the logistic regression model for younger patients, the preference for “quality of life” over “prolongation of life” remained a relevant factor after adjusting for covariates. Patients who stated that quality of life had been the decisive factor in weighing the treatment decision have a significantly higher chance of treatment deviating from guidelines compared to patients for whom quality of life and the prolongation of life were equally important (AOR: 2.08; 95% CI: 1.11–3.92). The strongest factor in the univariate analysis of younger patients—independent mobility—is no longer significant after adjustment for the covariates. In the elderly, the patient’s age at diagnosis remained positively associated with deviations from guideline recommendations (AOR: 1.06; 95% CI: 1.01–1.11). Likewise, the chance of receiving guideline-adherent treatment was lower in patients who were diagnosed with depression prior to their cancer diagnosis (AOR: 1.84; 95% CI: 1.00–3.40). In contrast, patients satisfied with the therapy decision-making process were more likely to be treated in accordance with guideline recommendations (AOR: 0.56; 95% CI: 0.34–0.91). The logistic regression models are displayed in Table 5.

**Table 5:** Multivariate logistic regression models by guideline adherence per age group

Characteristic	50 to 69 years			70 years and older		
	OR^1^	95% CI^1^	*P*	OR^1^	95% CI^1^	*P*
Mobility	0.39	0.12, 1.29	0.123			
Confidant available	0.86	0.42, 1.75	0.672			
Prolongation vs quality of life?						
Both equally important	–	–				
Prolongation of life	0.78	0.30, 2.05	0.617			
Quality of life	2.08	1.11, 3.92	**0.023**			
Knowledge of guidelines	0.67	0.41, 1.08	0.097			
Informed about illness and therapy	0.77	0.43, 1.40	0.393			
Psychological consultation offered	0.78	0.48, 1.27	0.316			
Age at diagnosis in years				1.06	1.01, 1.11	**0.021**
Depression				1.84	1.00, 3.40	**0.050**
Satisfied with decision-making				0.56	0.34, 0.91	**0.021**

^1^OR = Odds Ratio, CI = Confidence Interval

Bold *P* values (< .05) indicate statistical significance

The generalized variance inflation factor of the covariates was between 1.008 and 1.044 in the model for the younger patients and between 1.005 and 1.016 in the elderly patients’ model.

## Discussion

Using medical data and questionnaire responses from a sample of 1,150 women with breast cancer aged 50 to 91, we investigated potential age-related factors associated with adherence to guideline recommendations. Older women were considerably less likely to be treated per guideline recommendations than younger patients (75.4% overall adherence rate vs 86.4%). The association between age and guideline adherence was also found within the older age group, and is consistent with previous and comparable research (Wöckel et al. 2010; McDougall et al. 2021).

Deviations from guideline recommendations are required when this fits the patient’s individual situation and her needs and preferences. Accordingly, in the younger age group, we found that patients who reported that quality of life was guiding their therapy decision were twice as likely to be treated in deviation from guidelines compared to younger patients with no such preference. This suggests that in these cases, omissions of guideline-recommended therapy components were conscious, likely due to patients fearing their quality of life would be unduly impaired. Such seeming violations of the guidelines are not problematic when it is ensured that patients have made an informed decision with a balanced consideration of the expected side effects, including the availability of supportive care options (Jordan et al. 2017), but have not acted out of irrational fears.

On the other hand, in our study, both younger and elderly patients were more likely to be retrospectively convinced that their therapy was not appropriate when treated in the deviation of guideline recommendations. In these cases, the patients’ needs might not have been met sufficiently. This assumption is supported by two findings in the older age group: first, elderly patients with depression were significantly more likely to have received treatment deviating from guideline recommendations after adjustment for covariates. Depression in and of itself should not influence a treatment decision. However, it is conceivable that a depressive disorder impairs motivation for demanding guideline-based therapy (Hindmarch et al. 2013). All the more important is psycho-oncological counseling, which, according to the guidelines, should be offered to all patients. In our data, however, this was less often the case in older patients (68.1% compared to 46.2%). Second, elderly patients’ satisfaction with the decision-making process was significantly associated with adherence to guideline recommendations in univariate analysis and after adjustment for covariates. In cases where the patient’s personal situation suggests to deviate from guideline recommendations, pros and cons of the alternatives should be communicated and discussed particularly well. This in turn should be reflected in higher satisfaction with the decision-making process. The fact that this is not the case here is again an indication that patients’ needs have not been met sufficiently.

In the previous studies, a lack of social support and inclusion has been identified to inhibit successful cancer treatment (Kroenke et al. 2006; Lambert et al. 2018). This is not reflected by higher rates of guideline-discordant therapy in our sample; the level of social support was not related to the treatment decision in either younger or older people.

Research from the broader context of this study suggests that in elderly patients, informed and autonomous therapy decision-making should not be taken for granted due to those patients’ declining independence and subsequent dependence on third parties, like confidants and caregivers (Gieseler et al. 2021; Heidenreich et al. 2023). In Germany, for example, it has been criticized that cancer treatment is too seldom organized routinely by a managing or guiding physician (Pfaff and Schulte 2012). Then, the challenging task of coordinating the complex treatment process falls to the patient. We have not assessed the skills required for this in detail. However, it seems likely that those are less pronounced in older people. In our study, we found that the chance of receiving guideline-discordant care increased by 6% for each year of age. To some extent, the influence of age may represent a decline in communication and organizational skills. Future investigations that take a more differentiated look at the therapy decision-making process and allow for minute reconstruction of the reasons underlying therapy decisions are necessary here: which decisions are informed by the patients’ explicit wishes, and which treatment options were presented and recommended as allegedly suitable for their situation? Only in this way can a meaningful distinction be made between informed and autonomous decisions and those that either presume the patient’s will or do not even consider it.

### Strengths and limitations

Out of the many recommendations given in the guideline used, we selected a set of recommendations that are highly clinically relevant and could be adequately assessed with the available high-quality data on cancer and therapies that have undergone established structured registration and validation processes of clinical epidemiological cancer registries. Our comprehensive questionnaire addresses various factors related to the context of treatment decision-making in addition to clinical and psychosocial parameters. The response rate was high, indicating that our study sample corresponds to the target population.

Delays in reporting to cancer registries may have resulted in specific treatment components missing from patient records. As an overestimation of the actual deviation from guideline recommendations caused by incomplete data would affect younger and elderly patients similarly, comparisons between the groups are still permissible. To address potential issues with data completeness, we recorded therapy events with the questionnaire and supplemented the medical data with the corresponding answers, if necessary. The alignment between self-reported treatment data by patients with breast cancer with medical records was found to be high (Kool et al. 2018). However, uncertainties in patient-reported data regarding reliability remain. The same is true with regard to judgments and experiences, especially if the response interval is long. On average, the questionnaires were completed 79 weeks after the cancer diagnosis. Due to this time lag, there are probably many patients for whom the therapy succeeded and who have largely overcome their illnesses.

Although we carried out pre-tests, we must finally assume that questions have not been understood by all participants as intended. This applies, for example, to the differentiation between satisfaction with a decision-making process and its outcome.

### Conclusion

With our study, we tried to capture the complex events of therapy decisions of breast cancer patients. Several unanswered questions about highly personal and subjective reasons and implications are difficult to address with the conventional study designs, let alone to analyze adequately in terms of their complexity. As long as the context of the therapy decision-making regarding the patient’s knowledge and the autonomy of their decision is not clear, a deviation from guideline recommendations is not, in principle, a violation. This is especially true when the generalizability of a guideline recommendation to older patients is challenged by insufficient and inconclusive evidence, as is frequently the case (Desch et al. 2008; Wöckel et al. 2018b). Moreover, if the effectiveness of an intervention is questionable, but its side effects are certain to occur, the opposite may even be true. In light of these considerations, it is doubtful to what extent a guideline recommendation being followed should be regarded as a quality criterion in its own right. In clinical communication, attention must be paid to older patients for whom a deviation from the guidelines is being discussed to adequately address their needs and wishes. Our results suggest that this has not always been done adequately, especially for patients with depression.

## Data Availability

The datasets that support the findings of this study are available from the corresponding author upon reasonable request.
